# 547 One-Step Excision and Allograft in Burns: Uncommon Applications

**DOI:** 10.1093/jbcr/iraf019.176

**Published:** 2025-04-01

**Authors:** Christopher Fedor, Alexis Henderson, José Arellano, Mare Kaulakis, Hilary Liu, Garth Elias, Alain Corcos, Jenny Ziembicki, Francesco Egro

**Affiliations:** University of Pittsburgh School of Medicine; University of Pittsburgh School of Medicine; University of Pittsburgh Medical Center; University of Pittsburgh School of Medicine; University of Pittsburgh Medical Center; University of Pittsburgh Medical Center; University of Pittsburgh Medical Center; University of Pittsburgh Medical Center; University of Pittsburgh Medical Center

## Abstract

**Introduction:**

Cadaver allograft is essential in managing severe burn wounds, providing critical temporary coverage when immediate autografting is not possible. This technique is particularly important for deep burns that cannot heal independently, as it prevents wound desiccation and bacterial infection. In this study, we explore uncommon cases where excision and allograft were not followed by autografting, offering insights into wound healing.

**Methods:**

We conducted a retrospective review of patient records from January 2012 to January 2024 at an ABA-certified burn center, focusing on patients who underwent one-step excision and allograft without subsequent autografting. Data collected included patient demographics, burn characteristics (etiology, depth, total body surface area), surgical details related to allograft use for re-epithelialization, and post-operative outcomes. Follow-up data were recorded for an average of 6.7±13.5 months post-procedure.

**Results:**

27 patients (56% male, average age 50.9±25.1 years) met inclusion criteria. 26 burns were thermal, 1 chemical. 12 patients received palliative one-step excision and allografting, later passing away. 15 patients’ wounds re-epithelialized without further intervention. Of these 15, the median total body surface area of cutaneous burns was 6.5% (IQR: 3.5-15) and surgery occurred 6.3±7.8 days post-injury. Median allograft size was 247.5 cm2 (IQR: 100-978). Patients were instructed to trim the grafts as they lifted off, a natural process of graft rejection, to prevent interference with clothing or dressings. Full re-epithelialization occurred 34.0±12.8 days post-allograft. During the follow-up period, no hypertrophic scars or contractures were observed.

**Conclusions:**

This case series demonstrates an uncommon application of cadaveric allografts in burn management. Diverging from their typical use in wound bed preparation or temporary coverage, allografts were employed as a standalone treatment for two distinct purposes: (1) palliative care and (2) management of small burns with indeterminate depth and low risk for hypertrophic scarring or contracture. In these select cases, allografts successfully fulfilled their critical functions of preventing desiccation and stimulating vascularization, creating a temporary yet optimal environment for epithelialization. These findings may encourage burn surgeons to consider a one-step allograft application as a viable option in their treatment arsenal, particularly for select cases where traditional multi-stage procedures may be unnecessary or impractical.

**Applicability of Research to Practice:**

This one-step technique, suitable for select cases, offers potential clinical and economic benefits, challenging burn surgeons to consider more conservative methods that may improve outcomes while optimizing resource allocation and cost efficiency.

**Funding for the Study:**

N/A

